# Supplementary material for: Exploring the GM-CSF histidine triad as a modulator of structure, molecular motion, and ligand binding
Source: RSC Chem Biol. 2026 Jun 1;7(7):1409–19. doi: 10.1039/d6cb00019c (PMC13238453; doi:10.1039/d6cb00019c)
Supplement: CB-007-D6CB00019C-s001 [file CB-007-D6CB00019C-s001.pdf]

Supplementary Information (SI) for RSC Chemical Biology.
This journal is © The Royal Society of Chemistry 2026

Exploring the GM-CSF Histidine Triad as a Modulator of Structure, Molecular Motion, and Ligand Binding

Jennifer Y. Cui,[1#] Iz Varghese,[1,2#] Anna S. Bock,[1] Mariana Floody,[1] Fuming Zhang,[3] Brenda M. Rubenstein,[4,5,6] and George P. Lisi[1,7]*

[1]Department of Molecular Biology, Cell Biology, & Biochemistry, Brown University, Providence, RI USA
[2]Therapeutic Science Graduate Program, Brown University, Providence, RI USA
[3]Departments of Chemistry, Biology, & Chemical Biology & Engineering, Rensselaer Polytechnic Institute, Troy, NY USA
[4]Department of Chemistry, Brown University, Providence, RI USA
[5]Department of Physics, Brown University, Providence, RI USA
[6]Data Science Institute, Brown University, Providence, RI USA
[7]Giuliani RNA Center, Brown University Providence, RI USA

[#]Co-first authors
*Correspondence: george_lisi@brown.edu

Supporting Information

Figure S1. CD spectra of WT GM-CSF and variants
Figure S2. Effect of mutations on solvent accessibility.
Figure S3-S6. Relaxation parameters for WT GM-CSF and variants
Figure S7. Distance between residues 15 and 83 in MD Simulations.
Figure S8. Comparison of CSPs from HSQCs with MD-derived RMSF for WT GM-CSF and variants.
Figure S9. $^{1}H^{15}N$ HSQC NMR spectra and K_d curves of WT, H15Y, and H83Y with heparin (dp6) and ATP
Figure S10. Structural models of GM-CSF variants with either ATP (AlphaFold3) or Heparin (ClusPro).
Figure S11. CSP overlap analysis in GM-CSF titrations of ATP (orange) or dp6 (blue).
Figure S12. Representation of GM-CSF with hydrophobic and aromatic residues highlighted.
Figure S13. Solvent accessible surface area changes over time.

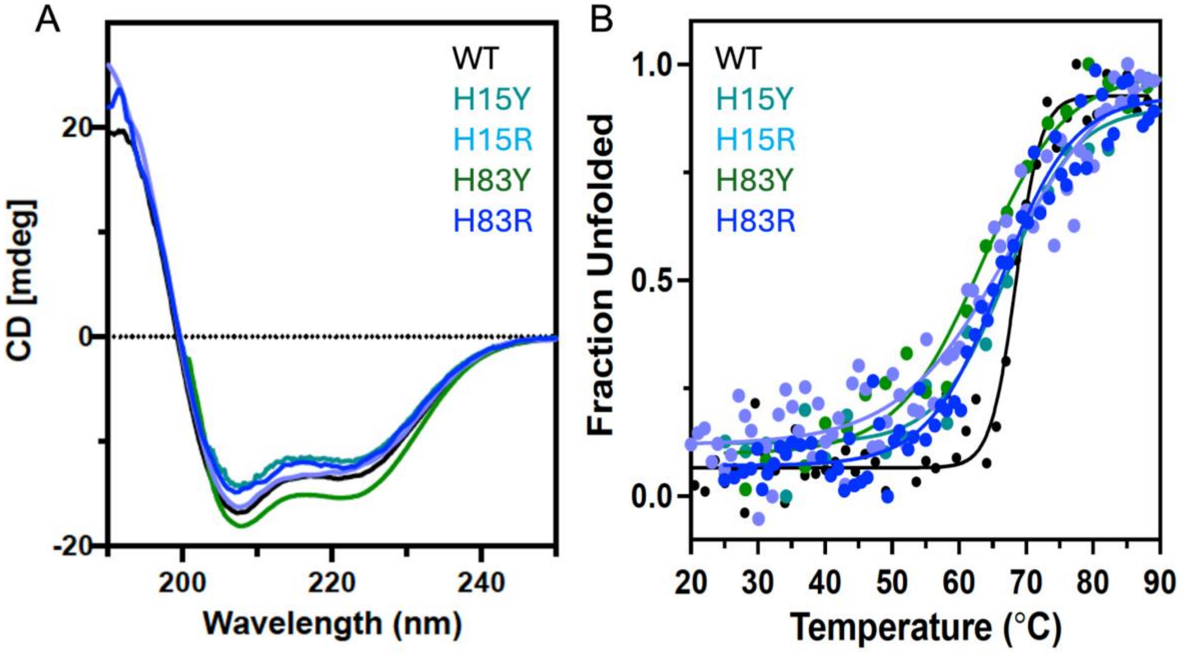

Figure S1. (A) CD spectra of WT (black), H15Y (cyan), H15R (light blue), H83Y (green), and H83R (blue) GM-CSF. Secondary structure profiles are highly similar. **(B)** Thermal denaturation experiments monitoring CD ellipticity at 220 nm for the same GM-CSF samples.

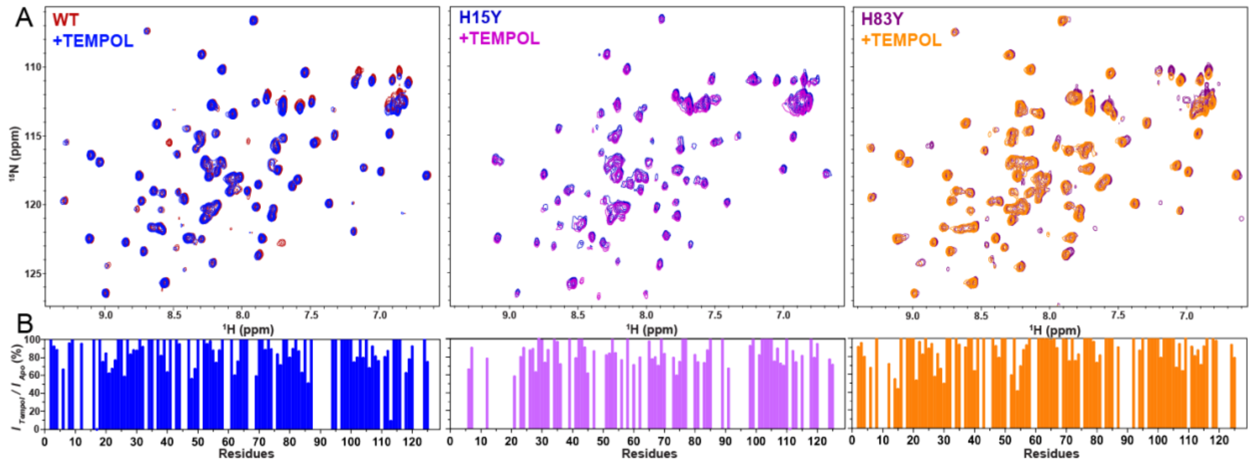

Figure S2. (A)^{1}H-^{15}N HSQC NMR spectral overlays comparing WT, H15Y, and H83Y GM-CSF without and saturated with 25 mM TEMPOL. The paramagnetic TEMPOL attenuates the signal of solvent-exposed hydrophobic residues. **(B)** Per-residue intensity ratios (I_{Tempol}/I_{Apo}) plotted as percent residual intensity, showing NMR signal attenuation at solvent-exposed regions.

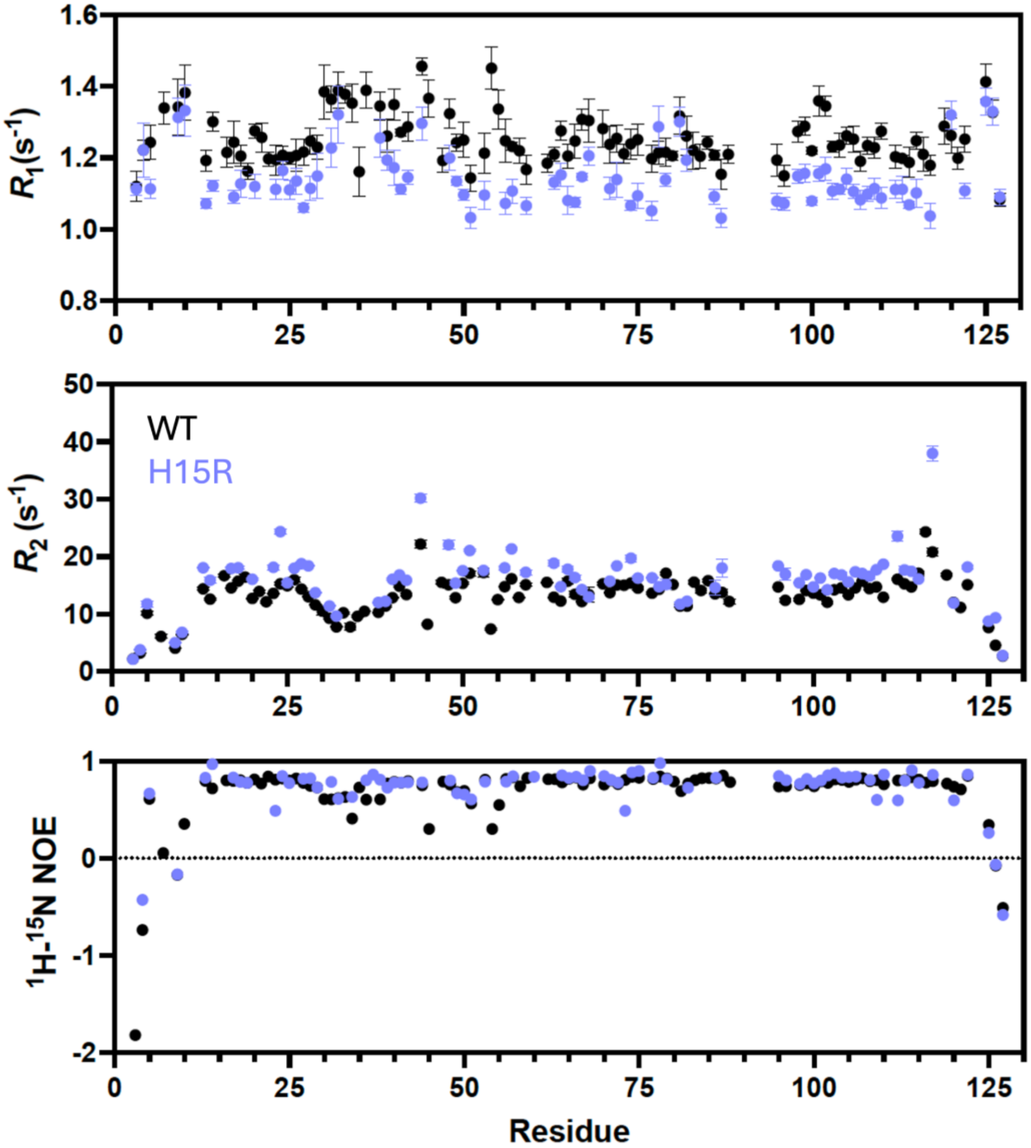

Figure S3. Per-residue R_1, R_2, and ^{1}H-[^{15}N] NOE relaxation parameters for WT GM-CSF (black) and H15R GM-CSF (light purple).

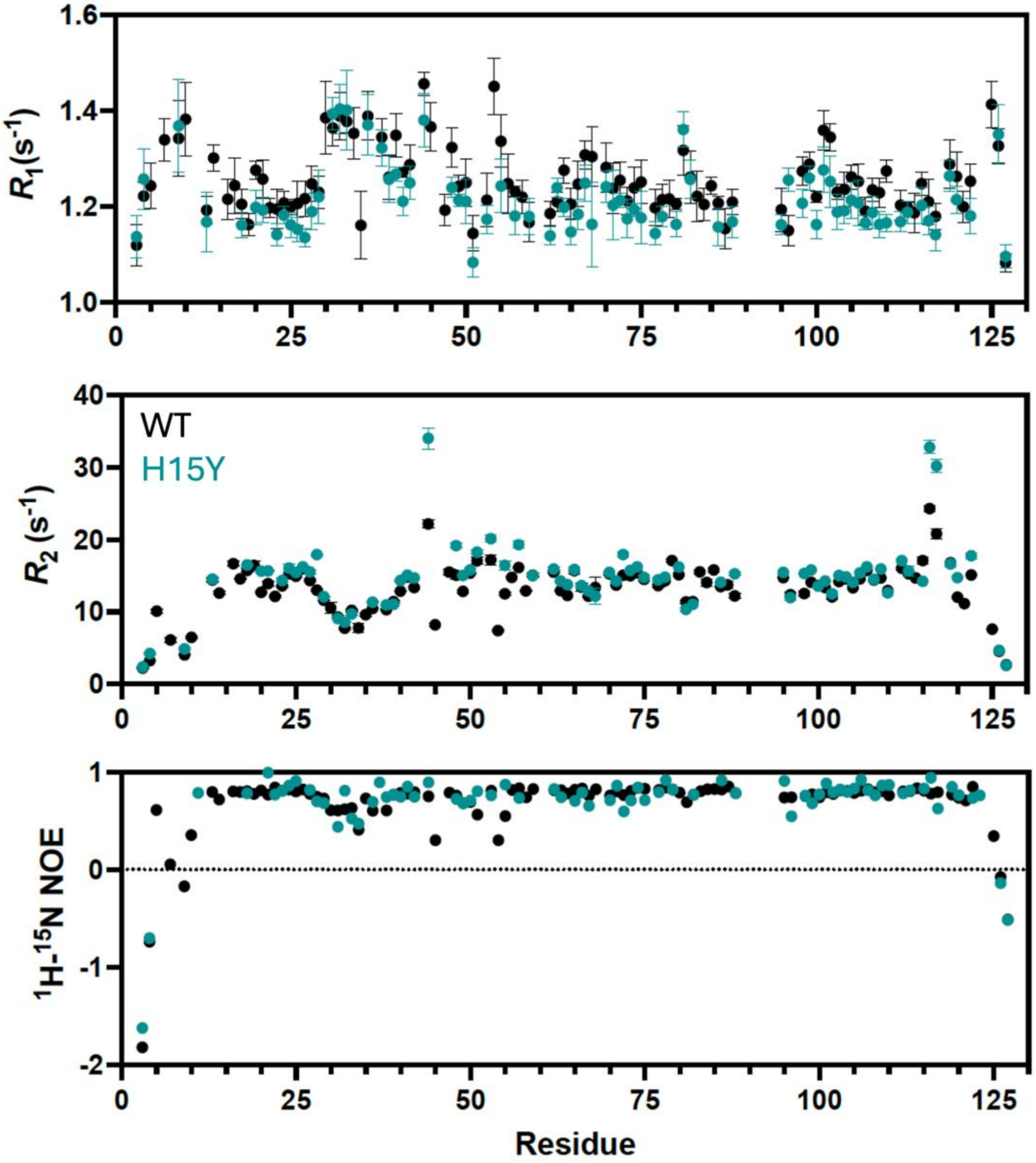

Figure S4. Per-residue R_1, R_2, and ^{1}H-[^{15}N] NOE relaxation parameters for WT GM-CSF (black) and H15Y GM-CSF (cyan).

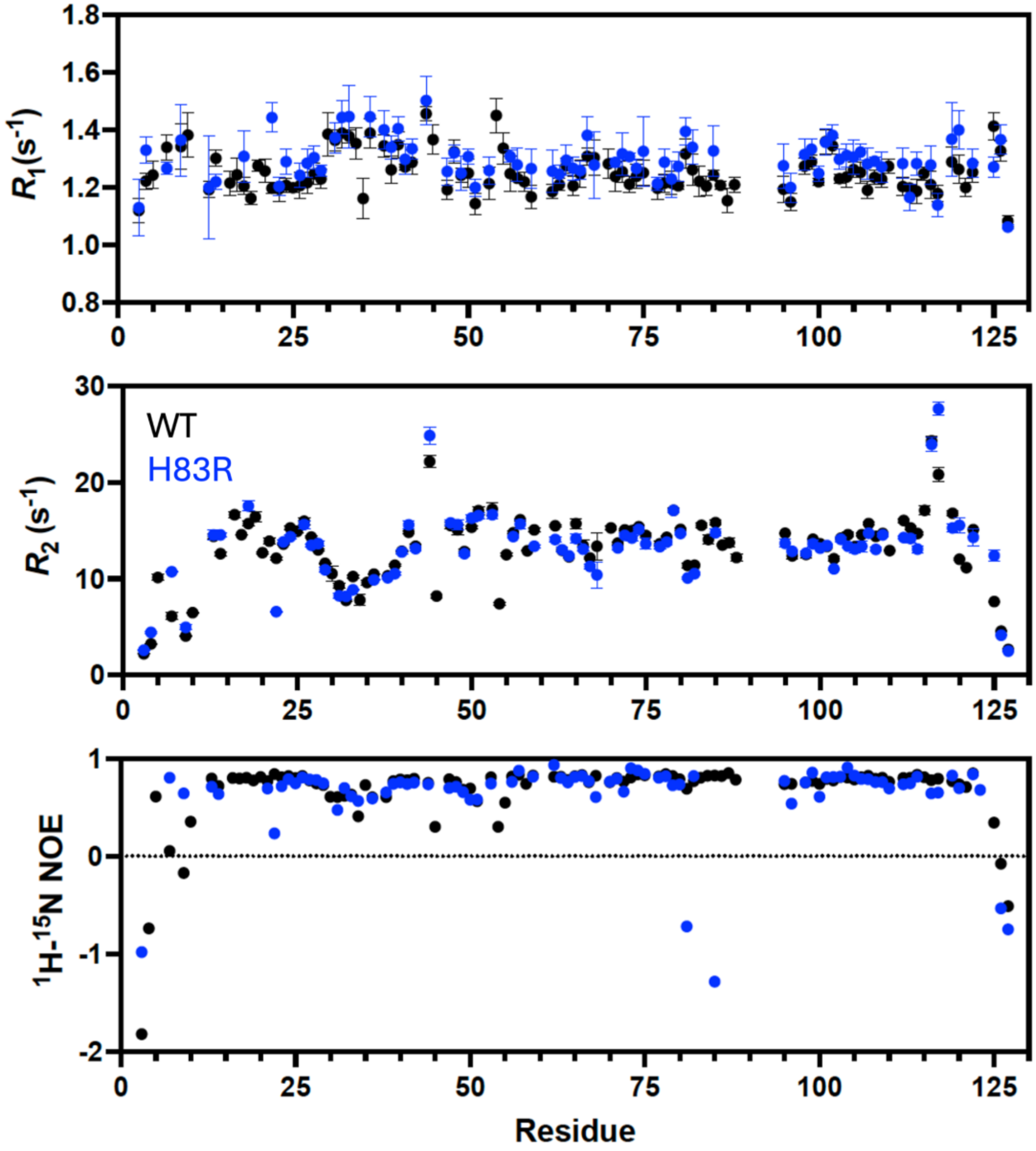

Figure S5. Per-residue R_1, R_2, and ^{1}H-[^{15}N] NOE relaxation parameters for WT GM-CSF (black) and H83R GM-CSF (blue).

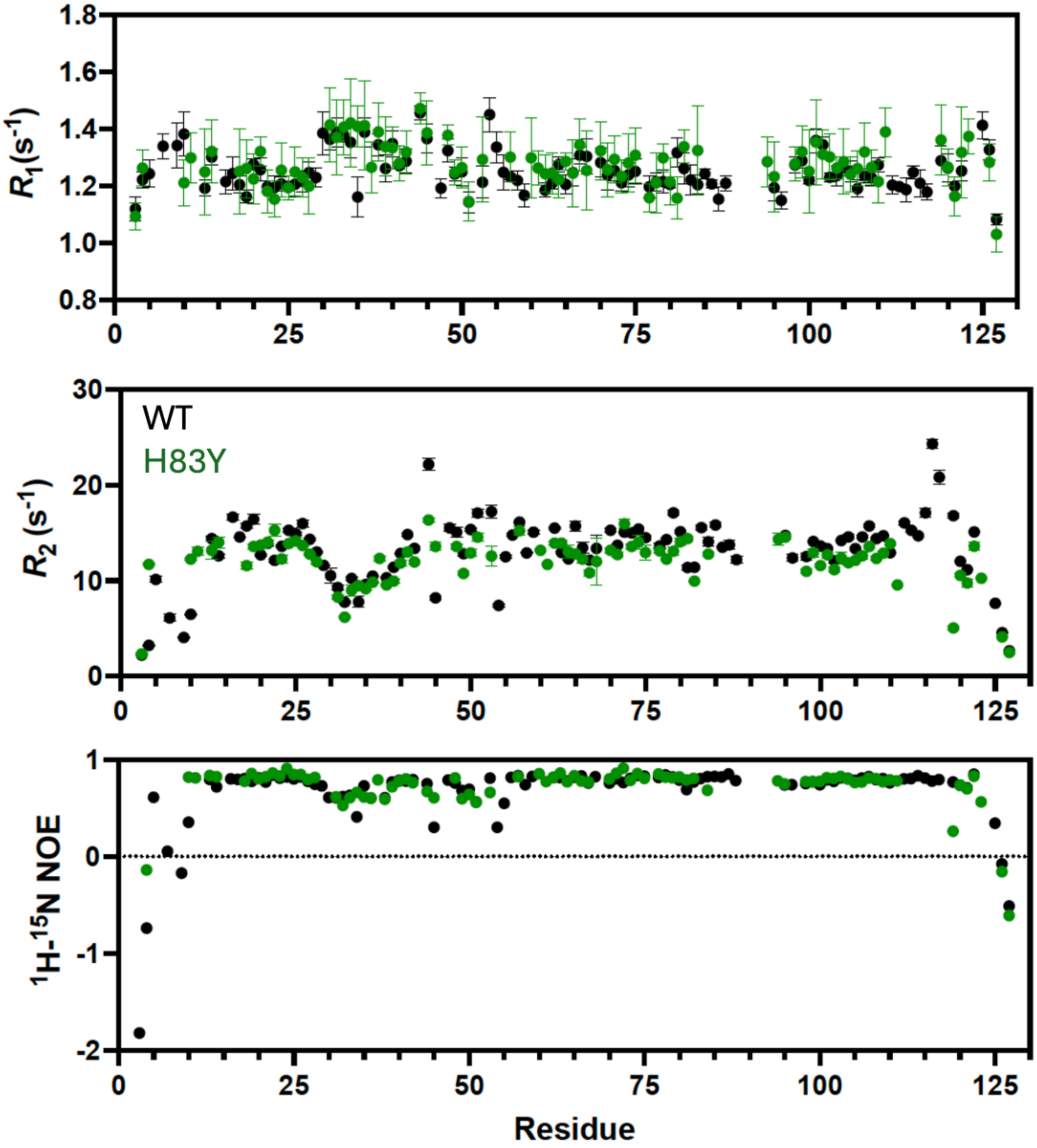

Figure S6. Per-residue R_1, R_2, and ^{1}H-[^{15}N] NOE relaxation parameters for WT GM-CSF (black) and H83Y GM-CSF (green).

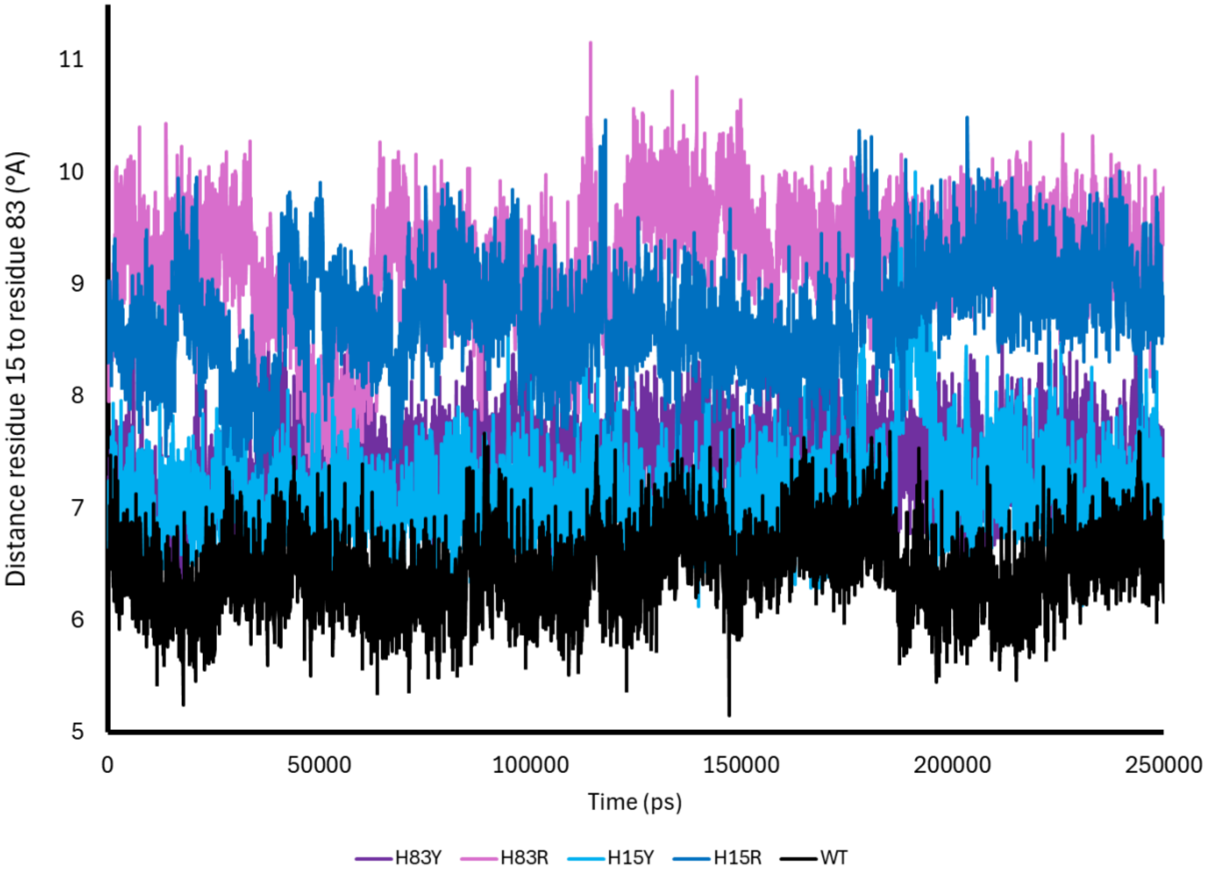

Figure S7. Distance between residues 15 and 83 in MD simulations over time.

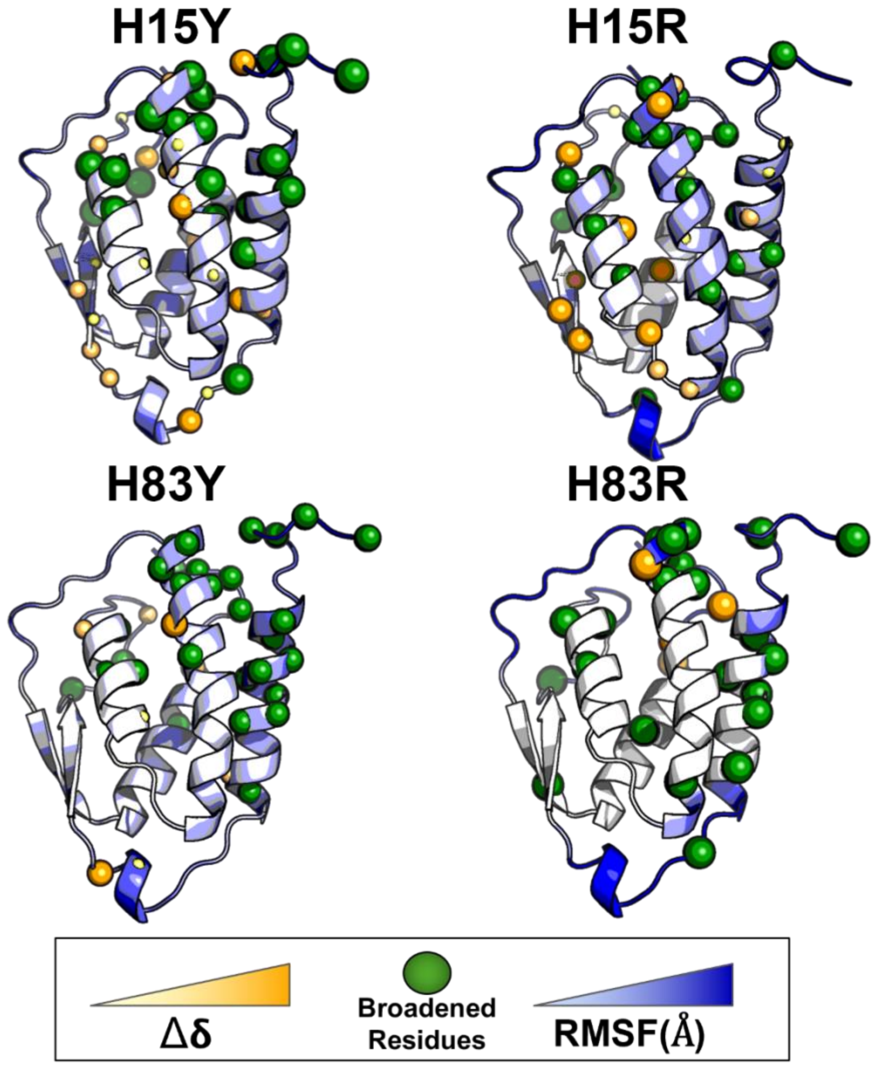

Figure S8. NMR CSPs (yellow) and broadened peaks (green) overlaid onto a structure mapped with relative RMSF (blue shades) demonstrate general agreement between residues experiencing heightened structural and dynamic changes in experiments and MD simulations. Increasing intensity of yellow and blue shades corresponds to increased magnitudes of CSPs and RMSF, respectively.

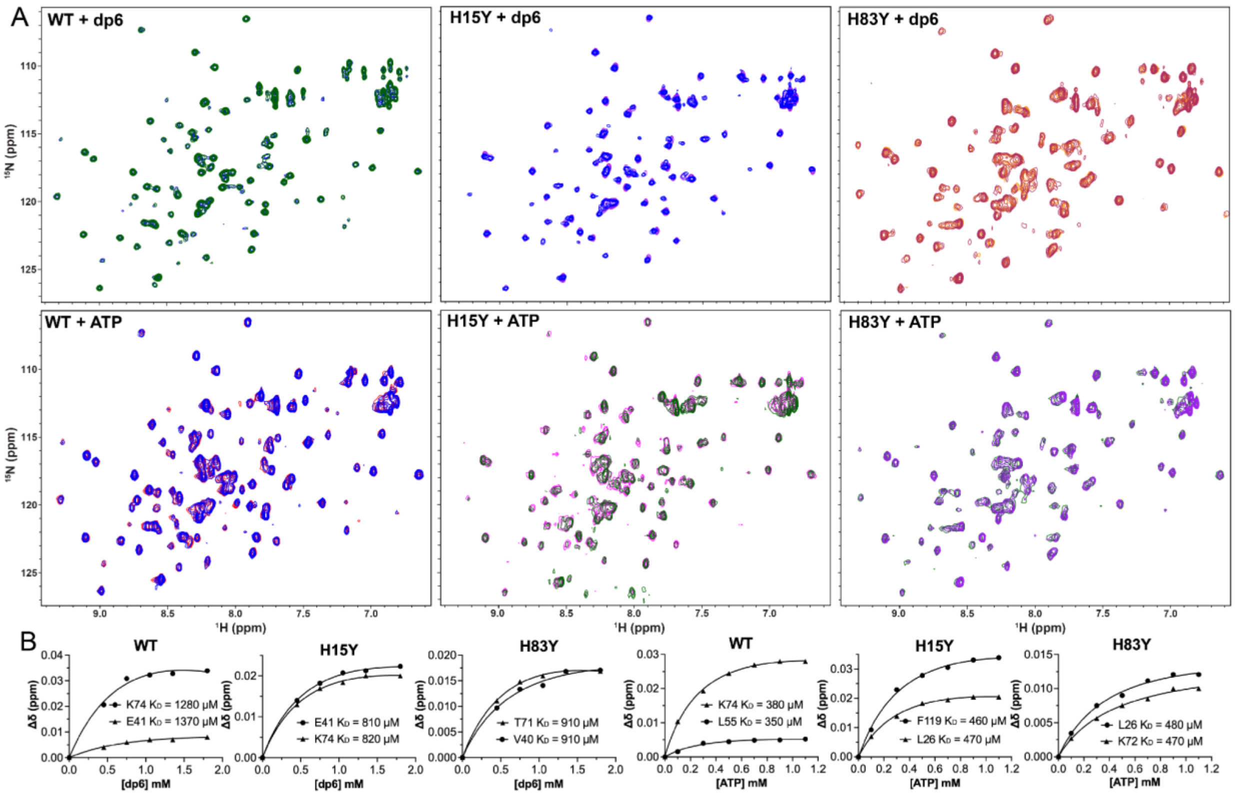

Figure S9. ^{1}H-^{15}N HSQC NMR spectra and K_d curves for WT, H15Y, and H83Y GM-CSF with heparin (dp6) and ATP. **(A)** ^{1}H^{15}N HSQC spectra overlay of WT, H15Y, and H83Y GM-CSF with 1.8 mM dp6 and 1.1 mM ATP. **(B)** K_d curve fits of ligand titration from representative peaks of each variant.

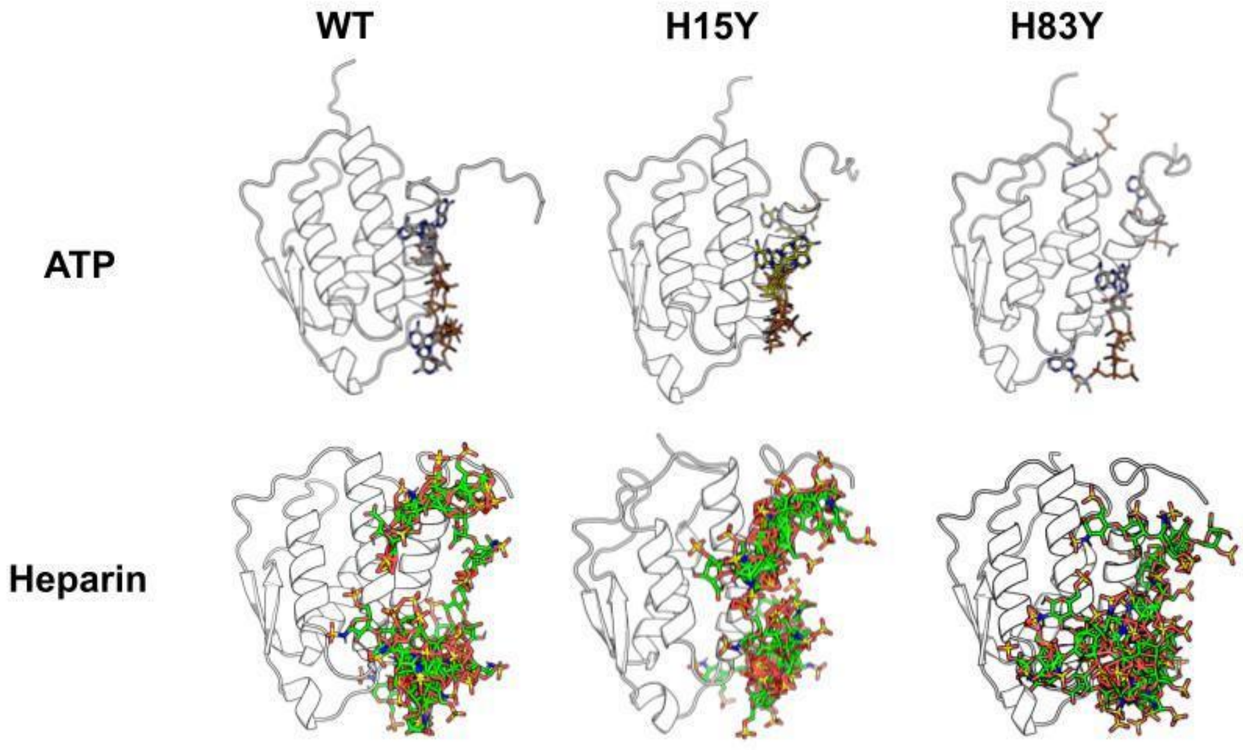

Figure S10. Structural models and predicted binding interactions of GM-CSF and variants with either ATP (AlphaFold3) or Heparin (ClusPro).

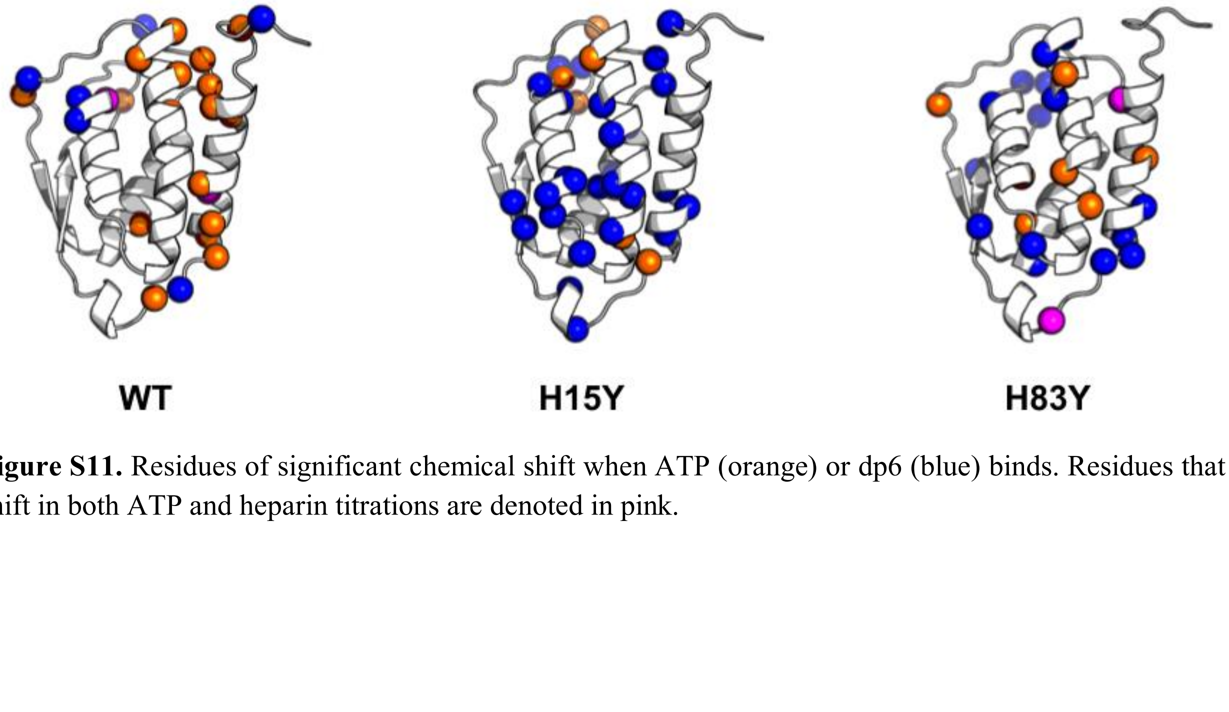

Figure S11. Residues of significant chemical shift when ATP (orange) or dp6 (blue) binds. Residues that shift in both ATP and heparin titrations are denoted in pink.

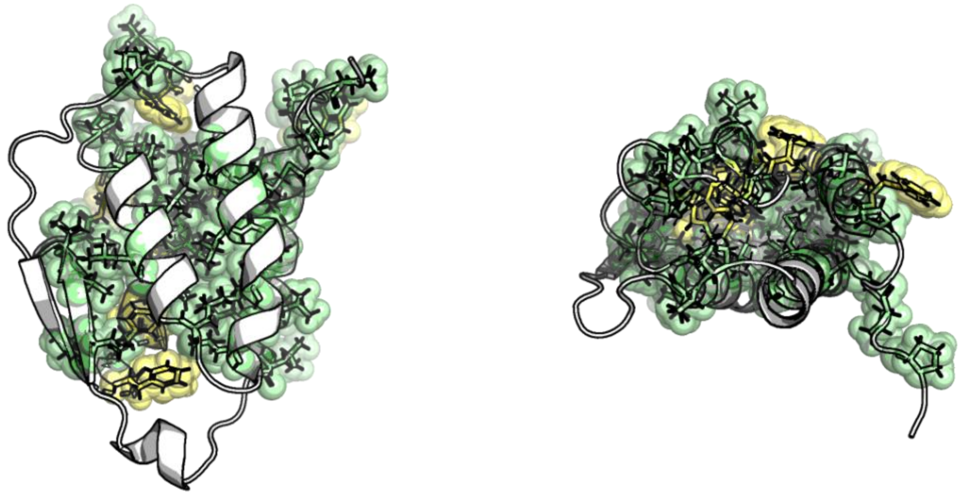

Figure S12. **Representation of GM-CSF with hydrophobic and aromatic residues highlighted.** Hydrophobic residues in the GM-CSF pocket are shown in green, while aromatic residues are shown in yellow.

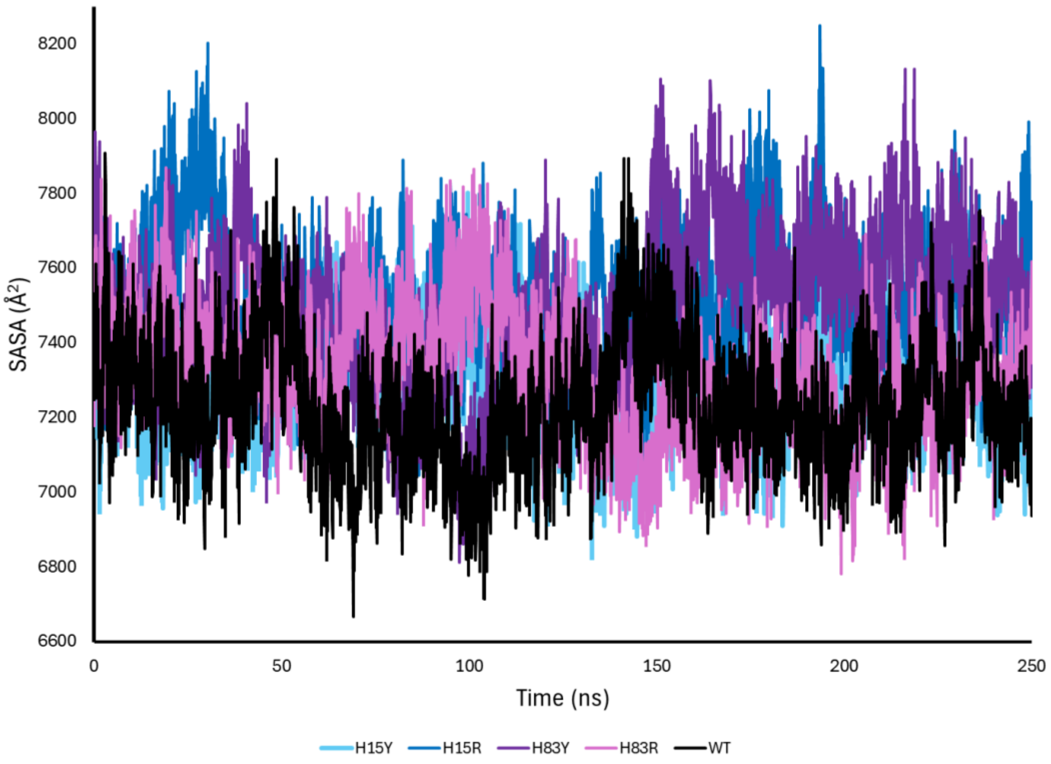

Figure S13. Solvent accessible surface area (SASA) changes over time for GM-CSF variants in MD simulations.
